# Needles for Metallic Sutures

**Published:** 1859-09

**Authors:** 


NEEDLE FOR METALLIC SUTURES.—Dr. Paul Goddard has recently devised a very simple and ingenious needle, which will be found to answer every purpose in the introduction of metallic sutures, thus obviating the necessity of twisting the wire, which renders its passage difficult.

The point of the needle is slightly curved and spear-shaped; its head is perforated at its centre with a hole of such a size as will accommodate the wire, and very near the head is an oblong eye, larger than in the ordinary needle. The wire is passed through the hole, coming out at the eye, and its extremity, doubled upon itself, is drawn back into the eye, thereby concealing the bent portion. The wire thus moves in the same line as the needle, and is passed without that jerking and pulling necessary when the ordinary needle is used. It can be obtained from Mr. Gemrig.

